# Added Therapeutic Benefits of Top‐Selling Drugs in Japan: A Cross‐Sectional Study Using Health Technology Assessment

**DOI:** 10.1111/cts.70243

**Published:** 2025-05-26

**Authors:** Hayase Hakariya, Akihiko Ozaki, Takanao Hashimoto, Frank Moriarty, Hideki Maeda, Tetsuya Tanimoto

**Affiliations:** ^1^ Interfaculty Institute of Biochemistry University of Tuebingen Tuebingen Germany; ^2^ Institute for Pharmaceutical and Social Health Sciences Ise Japan; ^3^ Breast and Thyroid Center Jyoban Hospital of Tokiwa Foundation Iwaki Fukushima Japan; ^4^ Kenkodo Pharmacy Osaki Miyagi Japan; ^5^ School of Pharmacy and Biomolecular Sciences RCSI University of Medicine and Health Sciences Dublin Ireland; ^6^ Regulatory Science, School of Pharmacy Meiji Pharmaceutical University Tokyo Japan; ^7^ Navitas Clinic Tokyo Japan

**Keywords:** added therapeutic benefit, health technology assessment, Japan, top‐selling drugs

## Abstract

It is unclear whether Japanese top‐selling drugs have meaningful added therapeutic benefits to justify their high sales. This question is relevant as Japan's healthcare costs are rising consistently, particularly due to increasing drug prices. This cross‐sectional study evaluated the added therapeutic benefits of Japan's top‐selling drugs in 2021 using ratings from established health technology assessment (HTA) agencies in Canada, France, and Germany. Drug characteristics and benefit ratings were obtained from public databases and HTA agencies, following the established method. Overall, added therapeutic benefit ratings were categorized as binary (high or low). Of 51 identified top‐selling drugs in Japan, 43 (86%) had at least one rating from three agencies. Notably, 20 (47%) received low added therapeutic benefit ratings even in our optimistic scenario. Low ratings were more common among small‐molecule drugs 15/20 (75%), while high ratings were predominant among biologics 14/23 (61%). Oncology drugs represented the largest category in both high 9/23 (39%) and low 5/20 (25%) groups. Interestingly, 9 drugs (9/16; 56%) approved between 2011 and 2021 received low ratings, compared to 41% (11/27) of those approved before 2011. Additionally, 70% of high‐benefit drugs received at least one expedited review, whereas this was 35% for low‐benefit drugs. Our findings revealed that many top‐selling drugs in Japan had low added therapeutic benefits. Utilizing HTA evaluation frameworks provides valuable insights, particularly in prioritizing drugs based on added therapeutic benefits. While full implementation of such a system in Japan requires further consideration, strengthening HTA processes could help ensure sustainable healthcare costs.

**Summary**

- What is the current knowledge on the topic?
  ○ Advancements in biologics, genetic therapies, and novel drug targets have transformed drug discovery. However, improvements in health outcomes have not kept pace, necessitating an increased focus on assessing added therapeutic benefit for decision‐making regarding drug pricing and reimbursement. Countries like Canada, France, and Germany conduct independent health technology assessments (HTA) to determine therapeutic value, while Japan recently introduced its own HTA agency (C2H) but has yet to integrate benefit ratings into pricing decisions.
- What question did this study address?
  ○ It is unclear whether Japanese top‐selling drugs have meaningful added therapeutic benefits to justify their high sales. This cross‐sectional study examined the added therapeutic benefit ratings of Japan's top‐selling drugs in 2021 as assessed by using ratings from established HTA agencies in Canada, France, and Germany.
- What does this study add to our knowledge?
  ○ Our findings revealed that 47%–81% of Japan's top‐selling drugs were rated as having low added therapeutic benefit, consistent with trends in the US. Biologics were more likely to receive high ratings, while many small‐molecule drugs provided limited benefits over alternatives. Additionally, discrepancies among HTA agencies highlight challenges in standardizing benefit assessments.
- How might this change clinical pharmacology or translational science?
  ○ Japan's HTA system could be strengthened to inform pricing decisions and promote cost‐effectiveness without discouraging innovation. Incorporating added therapeutic benefits into reimbursement frameworks may enhance healthcare sustainability while ensuring that patients receive valuable treatments.

## 1 Introduction

In recent decades, dramatic advancements in scientific technologies, such as diverse biologics, genetic therapies, and newly discovered drug targets, have contributed to drug discovery and development. Despite these advances, health outcomes have not seen corresponding improvements [1, 2]. Consequently, the importance of added therapeutic benefit has been emphasized in decision‐making regarding drug funding and pricing, as a means of managing increasing drug prices [3]. For instance, Canada, France, and Germany have their own independent health technology assessment (HTA) organizations, which assess the effectiveness and safety of drugs compared with existing treatments. These benefit ratings are leveraged in price negotiations for newly approved drugs [4, 5, 6, 7, 8], not to exceed the alternative treatments [9, 10, 11]. On the other hand, in the United States (US), which does not have a national HTA agency, many top‐selling medications have low added therapeutic benefits as rated by other nations' agencies [12].

In Japan, high‐cost drugs and drugs with clinically uncertain benefits have been documented, especially in the field of emerging biologics, such as regenerative medicine products and genetic medicines [13, 14, 15]. One scenario that can lead to such cases is the conditional/time‐limited early approval (CEA) of innovative drugs [16, 17]. This expedited approval pathway was originally introduced in regenerative medicine products in 2014 and expanded its program scope to include medical drugs in 2017. The program has enabled Japan's regulator to grant approval to drug candidates based on limited evidence, such as from single‐arm and non‐randomized trials, pending confirmatory trials to be conducted within a specific time limit. Although the CEA pathway is intended for serious conditions with limited existing treatments and only five drugs have been approved through the system as of June 2022, the presence and proliferation of such products with uncertain benefits on the market reinforce the necessity for a fair comparison with at least the limited existing treatments to assess the added therapeutic benefit.

To address high pharmaceutical expenditure, Japan recently established its own HTA agency (Core2 Health: C2H) in 2019 [18]. C2H evaluates medical devices or drugs with predicted peak market size beyond 5 billion Japanese yen (≒31.7 million USD) per year, as such products heavily affect the healthcare budget. However, as few drugs have had their added therapeutic benefits assessed to date, Japan's pricing agency does not take their assessments into consideration in the pricing process. Thus, taking advantage of added therapeutic benefit ratings by HTA agencies in other industrialized countries may inform decision‐making in Japan regarding which drugs provide limited additional benefits over alternative treatment options. Herein, we investigated Japan's 50 top‐selling drugs in 2021 and evaluated the added therapeutic benefit ratings of these drugs by HTA agencies in Canada, France, and Germany.

## 2 Materials and Methods

### 2.1 Top‐Selling Drugs in Japan

In this cross‐sectional study, we identified the 50 top‐selling drugs using publicly available Japanese domestic drug sales data for the 2021 fiscal year (spanning from April 2021 to March 2022), which has been reported by Japan's medical media (AnswersNews) based on IQVIA's market survey [19]. The survey covered medical drugs, biosimilars, and authorized generics with annual sales of more than 5 billion Japanese yen (31 million USD). However, no biosimilars or authorized generics were listed among the 50 top‐selling drugs.

### 2.2 Characterization of Drugs

We evaluated publicly available review reports from Japan's Pharmaceutical and Medical Device Agency (PMDA) to extract information about each drug [20], including the date of approval, drug type (small molecule or biologic), application category (new molecular entity or new indication; in the case of drugs with multiple indications, they may qualify for both categories), and to identify whether drugs qualified for any PMDA expedited review programs (Orphan drug designation, Sakigake, Yusen, and Jinsoku; drugs may qualify for multiple programs) [21, 22, 23, 24]. To further characterize each drug, we referenced the KEGG (Kyoto Encyclopedia of Genes and Genomes) BRITE database [25] to identify the World Health Organization's Anatomical Therapeutic Chemical (ATC) codes. Based on the assigned ATC codes [26], drugs were classified into one of ten therapeutic categories: COVID‐19, cardiovascular, endocrine, gastrointestinal, hematology, immunologic, oncology, ophthalmic, orthopedic, and other.

### 2.3 Therapeutic Benefit Ratings

We followed established methods to identify added therapeutic benefit ratings of drugs [6, 12]. Briefly, we used databases from HTA agencies in Canada (Human Drug Advisory Panel), France (National Authority for Health), and Germany (Federal Joint Committee), as their ratings are publicly available and determined independently of a drug's price. These three countries are among the highest‐income countries, have large pharmaceutical markets, and the population health profiles are comparable to that of Japan [27]. Added therapeutic benefit ratings were extracted in June 2023 for Canada and France, and in December 2023 for Germany.

We defined binary (high and low) variables for each HTA agency's assessment to categorize our therapeutic rating system, as there was a variety of ratings used across countries. Ratings of moderate, considerable, significant, substantial, and important therapeutic value were defined as high and minor, slight, no, non‐quantifiable, and less added value as low (Table 1). In the case of a drug with multiple ratings for the same indication, the most recent ratings were used. In the case of a drug with multiple indications and different ratings, ratings against the drug's indication in the therapeutic category in Japan that were listed in the original survey were applied. We applied the most favorable rating among cases of the same indication with different subpopulations and dosage forms to provide each country's most generous assessment of a drug's added therapeutic value. If a drug was approved in Japan for two or more indications, we investigated as many ratings for these indications in each agency's database and applied the most favorable rating. Drugs with no ratings by any of the three HTA agencies were excluded. Subsequently, we defined an overall therapeutic benefit rating scale. As this practice involves a certain assumption, we assessed two scenarios to get overall benefit ratings for each drug. First, in the optimistic scenario, drugs with a high rating from at least one of the three agencies were categorized as high and otherwise as low. Whereas, as a sensitivity analysis, the pessimistic scenario considered drugs with a high rating from two or three agencies as high, otherwise as low.

**TABLE 1:** Categorization of added therapeutic benefit ratings in Canada, France, and Germany^a^.

	Canada	France	Germany
Agency	Human Drug Advisory Panel	Haute Autorité de Santé	Gemeinsamer Bundesausschuss
Categories of therapeutic added benefit ratings	High: substantial or moderate improvement	High: important, moderate added benefit	High: major or significant or considerable added benefit
	Low: slight or no improvement^b^	Low: minor, no added benefit	Low: minor, lesser, non‐quantifiable, no added benefit

^a^ Represented criteria are as of June 2023.

^b^ One drug (Panitumumab) was assessed based on pre‐2010 categorization, which resulted in Category3, providing moderate, little, or no therapeutic advantage over comparable medicines.

### 2.4 Data Collection and Analysis

Brand and generic names of drugs were searched through online databases provided by HTA agencies in Canada, France, and Germany [28, 29, 30]. French and German databases were translated into English using Google Translate. We took a descriptive approach to characterize and analyze the drugs included in our study. Data were analyzed using Microsoft Excel.

### 2.5 Ethics Statement

All data used in this study were publicly available and nonidentifiable. No patients were involved in this study. Therefore, no ethical approval was required.

## 3 Results

### 3.1 Characteristics of Identified Top‐Selling Drugs

We identified a total of 51 drugs among the 50 top‐selling drugs list, since Equa (vildagliptin) and EquMet (combination of vildagliptin and metformin hydrochloride) were combined together in one rank (Table S1). This list included one of five medical drugs approved through the CEA pathway in Japan (pembrolizumab). Among the 51, we identified 43 (84%) that had at least one added therapeutic benefit rating from the three HTA agencies. The major therapeutic categories of these drugs were oncology (14 [33%]), immunology (7 [16%]), and endocrinology (6 [14%]) (Table 2). Sixteen drugs (37%) had at least one approved indication with an orphan drug designation, and 23 (53%) were reviewed under at least one expedited program.

**TABLE 2:** Characteristics of Japan's domestic top‐selling brand‐name drugs in the fiscal year 2021 by overall added therapeutic benefit rating (*n* = 43).

Characteristic	Overall added therapeutic benefit rating of drugs, no. (%)^f^			
	Optimistic scenario		Pessimistic scenario	
	High (*n* = 23)	Low (*n* = 20)	High (*n* = 8)	Low (*n* = 35)
Drug type				
Small molecule	9 (39)	15 (75)	3 (38)	21 (60)
Biologic	14 (61)	5 (25)	5 (63)	14 (40)
Therapeutic category				
COVID‐19	1 (4)	0	0	1 (3)
Cardiovascular	1 (4)	4 (20)	0	5 (14)
Endocrine	3 (13)	3 (15)	1 (13)	5 (14)
Gastrointestinal	0	1 (5)	0	1 (3)
Hematologic	2 (9)	0	1 (13)	1 (3)
Immunologic	4 (17)	3 (15)	0	7 (20)
Oncologic	9 (39)	5 (25)	5 (63)	9 (26)
Ophthalmic	1 (4)	1 (5)	1 (13)	1 (3)
Orthopedic	2 (9)	0	0	2 (6)
Other^a^	0	3 (15)	0	3 (9)
PMDA approval year				
Before 2011	16 (70)	11 (55)	0	17 (49)
2011–2021	7 (30)	9 (45)	8 (100)	18 (51)
PMDA expedited program^b^				
Any program	16 (70)	7 (35)	8 (100)	15 (43)
Orphan drug designation	10 (43)	6 (30)	5 (63)	11 (31)
Sakigake designation^c^	0	0	0	0
Priority review (Yusen)^d^	11 (48)	3 (15)	6 (75)	8 (23)
Rapid review (Jinsoku)^e^	2 (9)	1 (5)	0	3 (9)
New molecular entity	21 (91)	16 (80)	8 (100)	29 (83)
New indication	21 (91)	15 (75)	8 (100)	28 (80)

Abbreviation: PMDA, Japan's Pharmaceutical and Medical Device Agency.

^a^ Includes; duloxetine (Cymbalta), febuxostat (Feburic), and mirabegron (Tarlige).

^b^ Drugs may qualify for multiple PMDA's expedited programs.

^c^ The most prominent accelerated approval pathway in Japan, launched by MHLW in 2015, is similar to the Breakthrough Therapy Designation in the US and Priority Medicines in the EU.

^d^ Launched in 1993, similar to the fast track designation in the US. In addition to the drug candidates targeting serious diseases with no standard therapy, all the orphan‐designated products are applicable to this pathway.

^e^ Program unique to Japan. Drugs are specifically and individually designated to this pathway by Japan's Ministry of Health, Labour and Welfare.

^f^ In the optimistic scenario, drugs are considered to have a high added therapeutic benefit rating if at least one positive review in three HTA agencies' ratings, whereas the pessimistic scenario considers drugs with two or three positive reviews to be high.

Eight drugs without added therapeutic benefit ratings were listed and characterized in Table S2. None of them were reviewed under any expedited program by the PMDA.

### 3.2 Added Therapeutic Benefit Ratings

Among the top‐selling 51 drugs, HTA agencies in Canada covered 31 (61%) of the added therapeutic benefit ratings, while France covered 39 (76%) and Germany covered 22 (41%) (Table S1). HTA agencies in Canada and France gave more top‐selling drugs low added therapeutic benefit ratings, with 24 (77%) in Canada and 26 (67%) in France. Whereas the German agency gave 9 (41%) a low added therapeutic benefit rating; however, as noted, Germany had assessed less than half of the top‐selling drugs. No drugs analyzed in this study have been evaluated by the Japanese HTA agency to date.

Overall, 10 drugs (20%) were evaluated by HTAs in one country, 19 (37%) in two countries, and 14 (27%) in all three countries (Table S1). Seven drugs (7/10; 70%) assessed in only one country received a low added therapeutic benefit rating. In the case of drugs assessed in two countries, 11 (11/19; 58%) were consistently rated as low added therapeutic benefit, 1 (1/19; 5%) was consistently rated as high added therapeutic benefit, and ratings were discordant for 7 (7/19; 37%) drugs. For drugs assessed in all three countries, 2 (2/14; 14%) were consistently rated as low added therapeutic benefit, 1 (1/14; 7%) was consistently rated as high added therapeutic benefit, and ratings were discordant for 11 (11/14; 79%) drugs.

Of note, even in the optimistic scenario, 20 out of 43 drugs with at least one rating (47%) were rated as low added therapeutic benefit in our overall rating (i.e., no agency gave a high rating). In the pessimistic scenario, 35 drugs had low ratings (35/43; 81%). Of the drugs with low added therapeutic benefit, most were small‐molecule drugs regardless of the scenario, (15 [75%] in the optimistic and 21[60%] in the pessimistic), whereas among high added therapeutic benefit drugs a majority were biologics (14 [61%] and 5 [63%]) (Table 2). In the optimistic scenario, major therapeutic categories among drugs with a low added therapeutic benefit rating were oncology (5 [25%]) and cardiovascular diseases (4 [20%]), whereas oncology (9 [39%]) and immunology (4 [17%]) were predominant among drugs with high ratings (Table 2). However, when we applied a pessimistic scenario, all immunologic drugs had low‐benefit ratings, indicating the need for cautious interpretation of this finding.

Among low‐rating drugs in the optimistic scenario, similar numbers were approved before and after 2011 (i.e., 11 vs. 9 respectively), whereas among high‐rating drugs, a majority were approved before 2011 (16, 70%) (Table 2). Conversely, high‐rating drugs were all approved after 2011 in the pessimistic scenario.

In the optimistic scenario, a total of 7 drugs (35%) with low added therapeutic benefit and 16 drugs (70%) with high added therapeutic benefit underwent at least one PMDA expedited review program, and results were similar in the pessimistic scenario.

## 4 Discussion

Our findings highlighted that among the top‐selling drugs in 2021 in Japan, most of the evaluated drugs (20/43; [47%] optimistic, and 35/43; [81%] pessimistic scenario) received low added therapeutic benefit ratings from HTA agencies in Canada, France, and Germany. This trend was consistent with the findings that 55% of the US's 50 top‐selling drugs had a low added therapeutic benefit rating [12]. Given reports that only 2%–31% of new drugs in France, Canada, Germany, and the US receive additional benefit ratings over existing therapies [31, 32], and that approximately 75% of ultra‐expensive drugs in 2018 in France, Canada, and Germany were rated as having low added therapeutic benefit [7], the observed ratings of top‐selling drugs in Japan may present a favorable or similar situation. Among drugs in Japan with a low added therapeutic benefit rating in the optimistic scenario, 11 (55%) were medications that have been on the market for more than a decade: i.e., bevacizumab, leuprorelin acetate, paclitaxel, epinastine hydrochloride, azilsartan, rivaroxaban, and others. Moreover, in the case of bevacizumab, the indication for human epidermal growth factor receptor 2 (HER2)‐negative breast cancer has been withdrawn from the US market since 2011, as overall survival was not improved in the post‐marketing clinical trial [13, 33, 34, 35]. Such drugs with no proven clinical benefit could be reassessed by the regulatory agency. Simultaneously, we underscore that the situation wherein many top‐selling drugs provide low or no added benefits over other treatment options already on the market should not be overlooked, given Japan's strained medical/healthcare budget [36].

In Japan, added therapeutic benefit is not a key factor for the successful marketing of innovative drugs. As context for the high percentage of top‐selling drugs with a low added therapeutic benefit rating in this study, the Japanese government does not require sponsors to demonstrate a drug's added therapeutic benefit to obtain market authorization, nor does Japan's pricing agency, the Central Social Insurance Medical Council, for pricing. HTA is conducted after drugs have already entered the market and is considered only for drug price revision. Therefore, sponsors have few incentives for pursuing and demonstrating added therapeutic benefits when developing new drugs, and they are not necessarily assessed in comparison with already existing treatment options as part of the approval or pricing process. This situation may result in many “me too” drugs—drugs within the same mechanistic class but developed later than the first approved case—on the market. Indeed, within the analyzed top‐selling drugs, there are some examples: vonoprazan fumarate was approved in 2014, whereas Japan's first approval of a proton pump inhibitor was in 2001; azilsartan was marketed as a 7th angiotensin receptor blocker in Japan; and three sodium glucose linked transporter 2 (SGLT2) inhibitors—dapagliflozin, empagliflozin, and ipragliflozin—and dipeptidyl peptidase‐4 (DPP‐4) inhibitors—vidagliptin, sitagliptin, and teneligliptin—ranked in the 50 top‐selling drugs, respectively. While these may offer alternative treatment options to patients, the proliferation of such drugs may prevent precious drug‐development resources from pursuing clinically valuable drugs for patients [37]. To address this and achieve effective HTA, legislative measures that encourage stakeholders to consider or pursue additional therapeutic benefits upon price negotiation or product introduction to clinical practice might be beneficial [3].

On the other hand, we note that assessing added therapeutic or clinical benefit is not a standardized nor an easy task. It is well documented that traditional cost‐effectiveness analyses tend to capture only part of the advantages that therapeutics offer [38]. For instance, the positive impact of improved health on a patient's ability to return to work holds benefits for both patients and society, which is usually not reflected in incremental cost‐effectiveness ratio calculations [39]. Moreover, approaches for HTAs are divergent across the agencies and organizations [40], and the added therapeutic value of pharmaceutical products is still a matter of debate in the field, with many measurement scales proposed [41]. Indeed, our results suggest disparities among HTA agencies, with 79% (11/14) of drugs rated in all three countries resulting in a discordant assessment. One reason is that these ratings normally take cultural and societal norms, which may differ among countries, into account [42]. Therefore, we propose measures for both Japan and international HTA agencies. First, Japan can consider reinforcing its own HTA agency's assessment function, given Japan's peculiarities such as cultural norms, disease prevalence, and available standard therapies. The agency is still immature, with only 29 drugs having been assessed for added therapeutic benefit as of July 2024, presumably because they assess drugs with only a predicted large market size, and due to its recent establishment in 2019 [18]. Expanding their engagement should be intended to sustain the universal health coverage without discouraging pharmaceutical innovation. Simultaneously, our result may suggest the opportunity for international harmonization or collaboration of HTA to better explain the observed international disparities across regions we have identified, as already suggested among European countries [43]. This is essential because regional disparities may cause heterogeneous access to drugs [44].

Among the five drugs approved through the CEA pathway, pembrolizumab was observed on the top‐selling drug lists we investigated, probably due to the multiple indications for this high‐cost cancer immunotherapeutic agent. The indication for Japanese CEA was for a locally advanced or metastatic microsatellite instability‐high (MSI‐High) cancer; an application for this indication was declined in France in April 2023, while the German HTA agency rated it with evidence of significant additional benefit. The international disparity highlights the ambiguity in the drug's clinical or added therapeutic benefit, indicating the need for Japan's careful evaluation within the timeframe of the CEA.

Eight drugs (16% out of 51; listed on Table S2) did not receive an added therapeutic benefit rating by any of the three agencies. This is presumably because many of these drugs are Japan's original products, i.e., vonoprazan fumarate, ipragliflozin, and mirogabalin besilate. Although some stakeholders in Japan advocate for such domestic products as a means of supporting Japan's pharmaceutical industry and economic growth [45], this creates a deficit in evidence of added therapeutic benefit since international HTAs cannot be used. Moreover, top‐selling drugs without a rating available include omega‐3 fatty acids formulations and ketoprofen‐based poultices, both of which are commercially available over the counter, and a proton pump inhibitor. Taking these facts into consideration, the added therapeutic benefit of the eight drugs may not be as high as there are alternative medications. In fact, none of them qualified for PMDA's expedited review pathway (Table S2). This indicates that our benefit rating results may underestimate the overall level of low added therapeutic benefit among the top‐selling drugs in Japan. Moreover, the fact that many of the local pharmaceutical products are only available in‐country and, as such, not subject to HTA assessment in other countries further highlights the necessity of Japan's own functional HTA assessment.

### 4.1 Limitations and Future Indications of This Study

This study has limitations. First, our study was limited to three HTA agencies, and our findings may differ if we had included other agencies, such as the National Institute for Health and Care Excellence (NICE) in the United Kingdom. However, we did not include this agency as the ratings affect the corresponding drugs' NHS coverage, while Japanese drug approval and the national insurance coverage are independent of the HTA ratings. Second, the data we used for identifying the 50 top‐selling drugs in Japan were largely based on the sponsor's financial presentation document. Global pharmaceutical companies do not report sales data from Japan for all their products. Therefore, such products are not reflected in the ranking. Third, the added benefit ratings were based on the latest assessments available as of June 11, 2023. Ratings are generally conducted around the time of the drug approval; however, many of the drugs evaluated in this study have been on the market for some time, indicating the possibility that some have been surpassed by newer therapeutic options. This could bias our results toward overestimating added therapeutic benefit. Additionally, our follow‐up for Canada's HTA agency website indicated it was no longer accessible as of June 2024. Fourth, our overall therapeutic benefit rating scale applied the most favorable rating across indications, subpopulations, and dosage forms. While this gives a more comprehensive approach (i.e., only assigning a low rating where this is clearly consensus), it does potentially bias our rating scale toward having high added therapeutic benefit ratings. Fifth, we did not observe a clear correlation between drugs' sales and their added therapeutic benefits. This suggests higher sales are driven by other factors, e.g., higher market size due to disease prevalence, currently available local Japan's standard care in Japan, etc. Further multi‐dimensional study might be warranted in this respect. Sixth, while we suggested that Japan reinforce its HTA function, careful consideration is required when referencing HTA criteria or decisions from other countries, given Japan's peculiarities, such as a relatively large number of local pharmaceutical products that are only available domestically and therefore not evaluated elsewhere. Lastly, we did not estimate the financial impacts on the national health insurance budget of these drugs' sales. Investigating such economic or financial impact would be of interest in the future.

## 5 Conclusion

Our study revealed that nearly half of Japan's top‐selling drugs in 2021 received low added therapeutic benefit ratings from established international HTA agencies, even in the optimistic scenario. This finding may highlight the need for Japan to reassess drug pricing processes. When alternative treatment options are available, Japan could take HTA into account for pricing to move toward better cost‐effectiveness. While full implementation of a comprehensive HTA system requires careful consideration, strengthening HTA processes could contribute to ensuring sustainable healthcare costs. C2H, a newly established Japanese HTA agency, could play a crucial role in this regard. However, the complexities in assessing added therapeutic value, as evidenced by discordant ratings among different HTA agencies, suggest that Japan should develop a robust, culturally appropriate framework for drug evaluation. This approach could ultimately help balance innovation, cost‐effectiveness, and patient outcomes in Japan's healthcare system.

## Author Contributions

All authors wrote the manuscript. Hayase Hakariya and Tetsuya Tanimoto designed the research. Hayase Hakariya performed the research. Takanao Hashimoto and Hayase Hakariya analyzed and interpreted these data. Hayase Hakariya wrote the original draft of the manuscript.

## Conflicts of Interest

T.T. receives personal fees from Medical Network Systems MNES Inc. and Bionics Inc., outside the submitted work. A.O. receives personal fees from MNES Inc., Becton, Dickinson and Company, Taiho Pharmaceutical Co. Ltd., and Kyowa Kirin Inc., outside the submitted work. All other authors declared no competing interests for this work.

## Supporting information


Tables S1–S2
