# Supplementary material for: Added Therapeutic Benefits of Top‐Selling Drugs in Japan: A Cross‐Sectional Study Using Health Technology Assessment
Source: Clin Transl Sci. 2025 May 26;18(6):e70243. doi: 10.1111/cts.70243 (PMC12105913; doi:10.1111/cts.70243)
Supplement: Supplementary file 1 — Tables S1–S2 [file CTS-18-e70243-s001.pdf]

Supplemental Material

Added Therapeutic Benefits of Top-Selling Drugs in Japan: A Cross-Sectional Study Using Health Technology Assessment

Hayase Hakariya[1,2*], Akihiko Ozaki[3], Takanao Hashimoto[4], Frank Moriarty[5], Hideki Maeda[6], Tetsuya Tsanimoto[7]

1; Interfaculty Institute of Biochemistry, University of Tuebingen, Tuebingen 72076, Germany
2; Institute for Pharmaceutical and Social Health Sciences, Ise, Japan
3; Breast and Thyroid Cancer, Jyoban Hospital of Tokiwa Foundation, Iwaki, Fukushima 972-8322, Japan
4; Kenkodo Pharmacy, Osaki, Miyagi, Japan.
5; School of Pharmacy and Biomolecular Sciences, RCSI University of Medicine and Health Sciences, Dublin, Ireland
6; Regulatory Science, School of Pharmacy, Meiji Pharmaceutical University, Tokyo, Japan
7; Navitas Clinic, Tokyo, 190-0023, Japan

ORCID, e-mail:
Hayase Hakariya : orcid.org/0000-0002-9121-4551, hayase.hakariya@uni-tuebingen.de
Akihiko Ozaki : orcid.org/0000-0003-4415-9657, ozakiakihiko@gmail.com
Takanao Hashimoto : orcid.org/0000-0003-2924-3391, pharm.hashi@gmail.com
Frank Moriarty : orcid.org/0000-0001-9838-3625, frankmoriarty@rcsi.ie
Hideki Maeda : orcid.org/0000-0002-0117-3961, maeda@my-pharm.ac.jp
Tetsuya Tanimoto : orcid.org/0000-0002-9818-8587, tetanimot@yahoo.co.jp

*To whom correspondence may be addressed.
Hayase Hakariya, Ph.D.
Interfaculty Institute of Biochemistry, University of Tuebingen, 72076 Tuebingen, Germany
TEL: +49 7071 29 75377
Email: hayase.hakariya@uni-tuebingen.de, haya.pha3@gmail.com (H.H.)

Supplemental Contents

Supplemental Tables 3

Table S1. Top 50 selling drugs in fiscal year 2021 with their added therapeutic benefit ratings in Japan (n=51). 3

Table S2. Characteristics of Japan's top selling drugs from April 2021 to March 2022, for which added value evaluation has not been identified 5

Supplemental Tables

Table S1. Top 50 selling drugs in fiscal year 2021 with their added therapeutic benefit ratings in Japan (n=51).

Rank	Name	Approval Year	Drug Type	Therapeutic Category	Overall Rating*		Added Therapeutic Benefit Rarting		
					Optimistic	Pescimistic	France	Canada	Germany
1	Pembrolizumab	2016	Biologic	oncology	High	High	High	low	High
2	Nivolumab	2014	Biologic	oncology	High	High	High	low	High
3	Osimertinib	2016	Small molecule	oncology	High	High	High	High	High
4	Vonoprazan Fumarate	2014	Small molecule	gastrointestinal	n.a.	n.a.	n.a.	n.a.	n.a.
5	Edoxaban Tosilate Hydrate	2011	Small molecule	cardiovascular	High	low	low	low	High
6	Esomeprazole Magnesium Hydrate	2011	Small molecule	gastrointestinal	low	low	low	n.a.	n.a.
7	Rivaroxaban	2011	Small molecule	cardiovascular	low	low	low	low	n.a.
8	Bevacizumab	2007	Biologic	oncology	low	low	low	n.a.	n.a.
9	Casirivimab, Imdevimab	2021	Biologic	COVID-19	High	low	n.a.	n.a.	High
10	Azilsartan	2011	Small molecule	cardiovascular	low	low	n.a.	low	n.a.
11	Tolvaptan	2010	Small molecule	cardiovascular	low	low	low	low	n.a.
12	Aflibercept	2012	Biologic	ophthalmic	High	High	High	High	low
13	Atezolizumab	2017	Biologic	oncology	High	High	High	low	High
14	Ramucirumab	2015	Biologic	oncology	High	low	low	low	High
15	Ustekinumab	2010	Biologic	immunologic	low	low	low	low	n.a.
16	Nintedanib Ethanesulfonate	2015	Small molecule	cardiovascular	low	low	low	low	low
17	Adalimumab	2008	Biologic	immunologic	High	low	High	low	n.a.
18	Duloxetine	2009	Small molecule	Other	low	low	low	n.a.	n.a.
19	Enzalutamide	2014	Small molecule	oncology	High	High	High	low	High
20	Golimumab	2011	Biologic	immunologic	low	low	low	low	n.a.
21	Tocilizumab	2005	Biologic	immunologic	High	low	High	low	n.a.
22	Emicizumab	2018	Biologic	Hematology	High	High	High	High	low
23	infliximab	2001	Biologic	immunologic	High	low	High	n.a.	n.a.
24	Febuxostat	2010	Small molecule	Other	low	low	low	low	n.a.
25	Tacrolimus Hydrate	2000	Small molecule	immunologic	High	low	High	low	n.a.
25	Teriparatide Acetate	2011	Biologic	orthopedic	n.a.	n.a.	n.a.	n.a.	n.a.
27	Denosumab	2013	Biologic	orthopedic	High	low	low	High	n.a.
28	Mirabegron	2011	Small molecule	Other	low	low	n.a.	low	low
28†	Vildagliptin†	2009	Small molecule	endocrine	low	low	low	n.a.	low
28†	Metformin Hydrochloride†	2015	Small molecule	endocrine	low	low	low	n.a.	low
30	Dapagliflozin Propylene Glycolate Hydrate	2014	Small molecule	endocrine	High	High	High	n.a.	High
31	Empagliflozin	2014	Small molecule	endocrine	low	low	low	low	low
32	Dulaglutide	2015	Biologic	endocrine	High	low	low	low	High
33	Pemetrexed Sodium Hydrate	2006	Small molecule	oncology	High	low	High	n.a.	n.a.
34	EPA・DHA formulation	2012	Small molecule	cardiovascular	n.a.	n.a.	n.a.	n.a.	n.a.
35	Pertuzumab	2013	Biologic	oncology	High	low	n.a.	High	low
35	Ketoprofen	2009	Small molecule	orthopedic	n.a.	n.a.	n.a.	n.a.	n.a.
37	Romosozumab	2018	Biologic	orthopedic	High	low	low	low	High
38	Ipragliflozin L-Proline	2013	Small molecule	endocrine	n.a.	n.a.	n.a.	n.a.	n.a.
39	Mirogabalin Besilate	2018	Small molecule	orthopedic	n.a.	n.a.	n.a.	n.a.	n.a.
39	Eltrombopag Olamine	2010	Small molecule	Hematology	High	low	low	High	n.a.
41	Pegfilgrastim	2014	Biologic	oncology	n.a.	n.a.	n.a.	n.a.	n.a.
42	Epinastine Hydrochloride	2004	Small molecule	immunologic	low	low	low	n.a.	n.a.
43	Paclitaxel	2009	Small molecule	oncology	low	low	low	n.a.	n.a.
44	Alectinib Hydrochloride	2014	Small molecule	oncology	low	low	low	n.a.	low
45	Leuprorelin Acetate	2002	Small molecule	oncology	low	low	low	n.a.	n.a.
46	Abemaciclib	2017	Small molecule	oncology	High	low	low	low	High
47	Ranibizumab	2009	Biologic	ophthalmic	low	low	low	low	n.a.
48	Panitumumab	2010	Biologic	oncology	low	low	low	low	n.a.
49	Sitagliptin Phosphate Hydrate	2009	Small molecule	endocrine	High	low	low	n.a.‡	High
50	Teneligliptin Hydrobromide Hydrate	2012	Small molecule	endocrine	n.a.	n.a.	n.a.	n.a.	n.a.

n.a. = Not Available

* In the optimistic scenario, drugs are considered to have a high added therapeutic benefit rating if at least one positive review in three HTA agencies' ratings, whereas the pessimistic scenario considers drugs with two or three positive reviews high.

† Total sales of Equa tablets (vitagliptin) and EquMet Combination Tablets LD (combination of vidagliptin and metformin hydrochloride) were counted as rank. However, since drug approvals are based on their components, we characterized both compounds respectively.

‡ Canada's health technology assessment agency reviewed the drug prior to 2010. Since the evaluation provided moderate, little or no therapeutic advantage over comparable medicines but it was not possible to discern moderate from little or no added benefit, the data was excluded from the analysis.

Table S2. Characteristics of Japan's top selling drugs from April 2021 to March 2022, for which added value evaluation has not been identified

Sales Rank	Name	Approval Year	Drug Type	Therapeutic Category	any expedited programs
4	Vonoprazan Fumarate	2014	Small molecule	gastrointestinal	No
25	Teriparatide Acetate	2011	Biologic	orthopedic	No
34	EPA・DHA formulation	2012	Small molecule	cardiovascular	No
35	Ketoprofen	2009	Small molecule	orthopedic	No
38	Ipragliflozin L-Proline	2013	Small molecule	endocrine	No
39	Mirogabalin Besilate	2018	Small molecule	orthopedic	No
41	Pegfilgrastim	2014	Biologic	oncology	No
50	Teneligliptin Hydrobromide Hydrate	2012	Small molecule	endocrine	No
